# The features of degenerative lumbar scoliosis in rheumatoid arthritis patients -matched cohort study

**DOI:** 10.1186/1748-7161-10-S1-P23

**Published:** 2015-01-19

**Authors:** Hirohisa Yagi, Hiroyuki Yasuda, Akinobu Suzuki, Akira Matsumura, Hidetomi Terai, Hiromitsu Toyoda, Sho Dohzono, Hiroaki Nakmura

**Affiliations:** 1 Osaka City University Graduate School of Medicine, Japan; 2 Osaka General Hospital of West Japan Railway Company, Japan; 3 Osaka City General Hospital, Japan

## Objective

The lumbar lesion in rheumatoid arthritis (RA) have been paid less attention, but some previous studies demonstrated the high prevalence of lumbar spondylolisthesis and lumbar scoliosis. The lumbar lesion accompanied with RA is often difficult to treat, and it is important to know the characteristics of lumbar lesion in RA patients. The purpose of this study is to clarify the features of lumbar scoliosis in RA patients compared with degenerative lumbar scoliosis in non-RA patients.

## Material and methods

A total of 54 patients (44 women and 10 men, 69.3 years, Cobb angle: 14.6 degrees) with scoliosis (Cobb angle of more than 10 degrees) who fulfilled the revised criteria of the American Rheumatism Association were included in this study. As control, age, sex, and Cobb angle matched 54 patients without RA were selected and also included. We evaluated superior/inferior end vertebra, apical vertebra and osteophyte formation using Nathan's classification (1-4) on plain X-rays. These parameters were compared between two groups using Man Whitney U-test.

## Results

The level of apical vertebra was significantly upper in RA than non-RA group. The level of superior end vertebra was also significantly upper in RA group, but there was no significant difference in the level of inferior end vertebra between two groups. The levels of curve was more wide in RA groups (RA group: 4.9 levels, non RA group: 3.6 levels, P value was less than 0.01). The degree of osteophyte formation was significantly greater in non RA group.

## Discussion

The present results showed the differences between lumbar scoliosis with RA and that without RA. These differences may indicate that the process or cause of scoliosis development in RA is different from that of degenerative scoliosis. Further, the less osteophyte formation may suggest that the lumbar scoliosis with RA is more likely to have instability, and these differences should be taken into consideration in the treatment of lumbar scoliosis with RA.

